# Malignant perineurioma derived from the retroperitoneum with an aggressive clinical course: a case report

**DOI:** 10.1186/s40792-024-01915-9

**Published:** 2024-05-13

**Authors:** Ken Kunugitani, Satoshi Ogiso, Masakazu Fujimoto, Tomoaki Yoh, Hisaya Shirai, Shinya Okumura, Hirofumi Hirao, Takamichi Ishii, Akihiko Yoshida, Etsuro Hatano

**Affiliations:** 1 Division of Hepato-Biliary-Pancreatic Surgery and Transplantation, Department of Surgery, Graduate School of Medicine, Kyoto University, 54 Shogo-in Kawahara-Cho, Sakyo-Ku, Kyoto, 606-8507 Japan; 2 Department of Diagnostic Pathology, Kyoto University Hospital, Kyoto, Japan; 3 Department of Surgery for Abdominal Oncology and Organ Regeneration, Graduate School of Medicine, Kyoto University, Kyoto, Japan; 4 Department of Diagnostic Pathology, National Cancer Center Hospital, Tokyo, Japan

**Keywords:** Cancer gene panel testing, Ectopic meningioma, Malignant perineurioma, Neurofibromatosis type 2 gene, Retroperitoneal sarcoma

## Abstract

**Background:**

Malignant perineurioma is a rare malignant counterpart of perineurioma derived from perineural cells. Resection is the primary option for the treatment of malignant perineuriomas; however, patients often develop recurrence after resection, and effective treatment for advanced or recurrent lesions needs to be established. This report describes a 51-year-old female with a rare malignant perineurioma in the retroperitoneum, which contributing valuable insights to the literature.

**Case presentation:**

The patient presented with abdominal distension and the imaging work-up revealed a huge hemorrhagic tumor in the retroperitoneum and obstruction of inferior vena cava by the tumor. The patient underwent surgery retrieving the tumor combined with left hemiliver and retrohepatic vena cava, which confirmed the diagnosis of a malignant perineurioma based on histopathological and immunohistochemical examination. Cancer gene panel testing identified mutations in *NF2*. Radiotherapy was administered for peritoneal dissemination 2 months after surgery, and the patient died from disease progression 6 months after surgery.

**Conclusions:**

This rare case highlights the challenges in managing retroperitoneal malignant perineuriomas. The aggressive characteristics and limited treatment options for advanced malignant perineuriomas underscore the need for understanding the pathogenesis and developing effective systemic therapies. The identification of an *NF2* mutation provides significant insights into potential therapeutic target.

## Background

Malignant perineurioma is a rare malignant counterpart of perineurioma derived from perineural cells. Histologically, it is characterized by hypercellularity, high mitotic count and necrosis [1]. The effective treatment has not been well established for malignant perineurioma, although primary treatment option is surgical resection [1]. There have been 24 cases of malignant perineurioma reported to date, most of which occurred in soft tissues of the trunk, followed by the upper and lower limbs, and 4 patients died of the tumor [2–6]. To the best of our knowledge, there have been only one report on malignant perineurioma originating from the retroperitoneum [7], and its clinicopathological characteristics remain unclear. Here, we report the detailed clinical course and genetic testing results of a second case of retroperitoneal malignant perineurioma that died 6 months after surgery.

## Case presentation

**Fig. 1:**
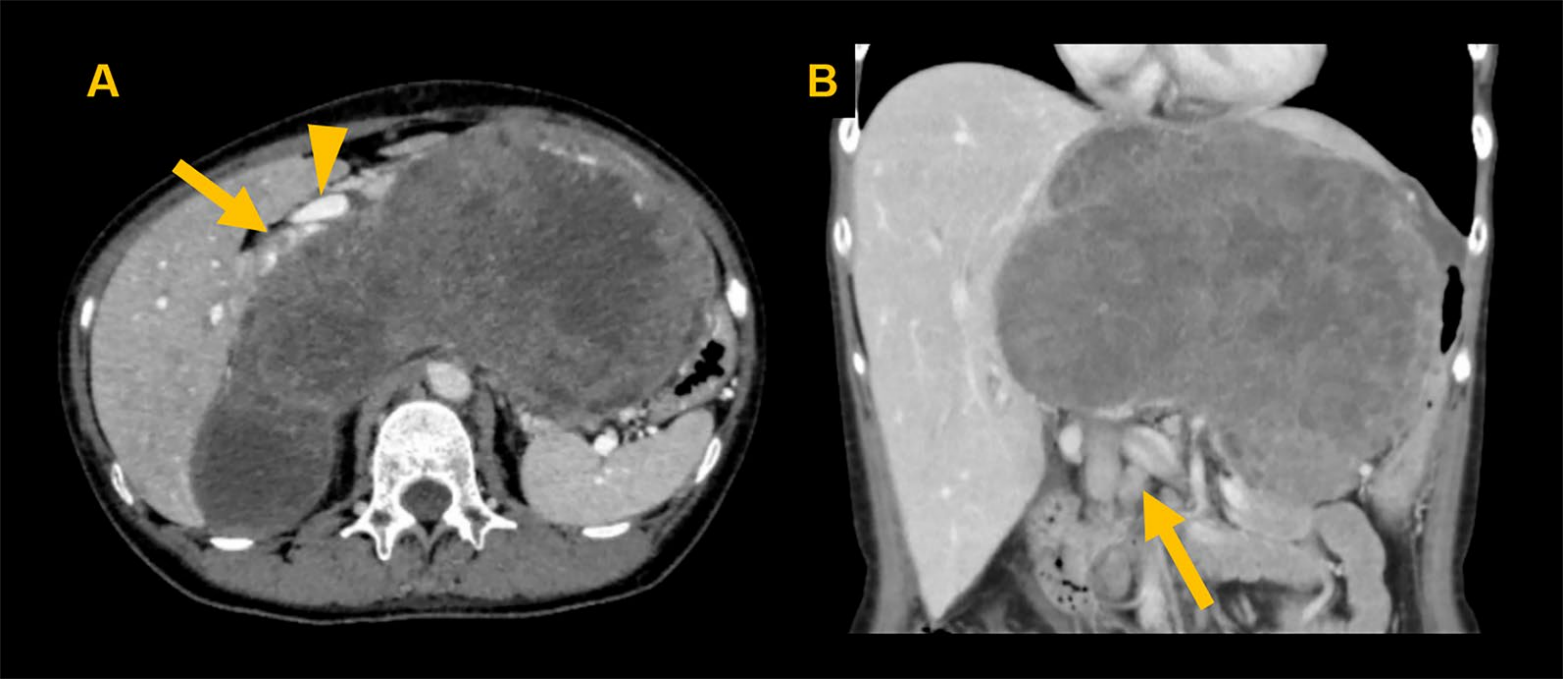
Preoperative imaging findings. Contrast-enhanced CT shows a hemorrhagic retroperitoneal tumor (22 × 9 × 15 cm) compressing the IVC (arrow) and portal vein (arrowhead) (**A**) and an enlarged para-aortic lymph node (arrow) (**B**)

**Fig. 2:**
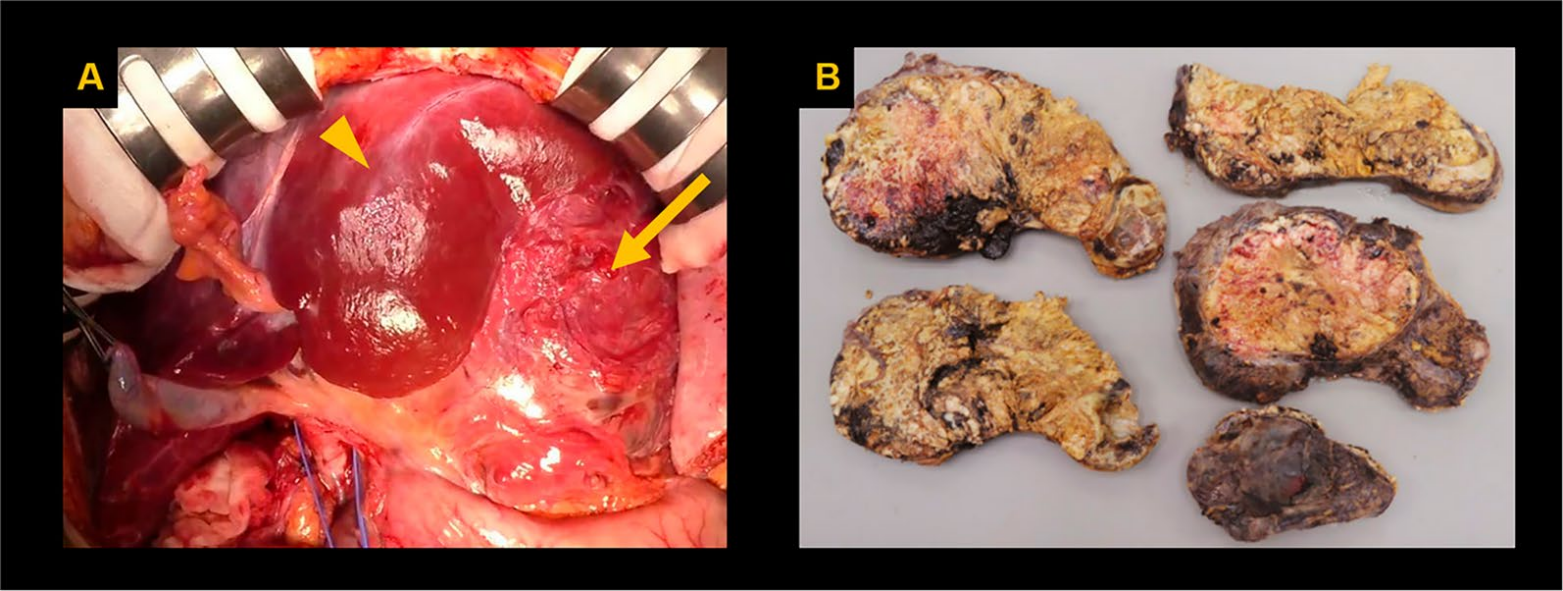
Intraoperative findings (**A**). The left lobe of the liver (arrowhead) and IVC were extensively adherent to the tumor (arrow) and were resected en bloc. The resected specimen (22 × 18 × 17.5 cm) was hemorrhaged and necrotic, with white lobulated nodules and edematous changes at the margins (**B**)

**Fig. 3:**
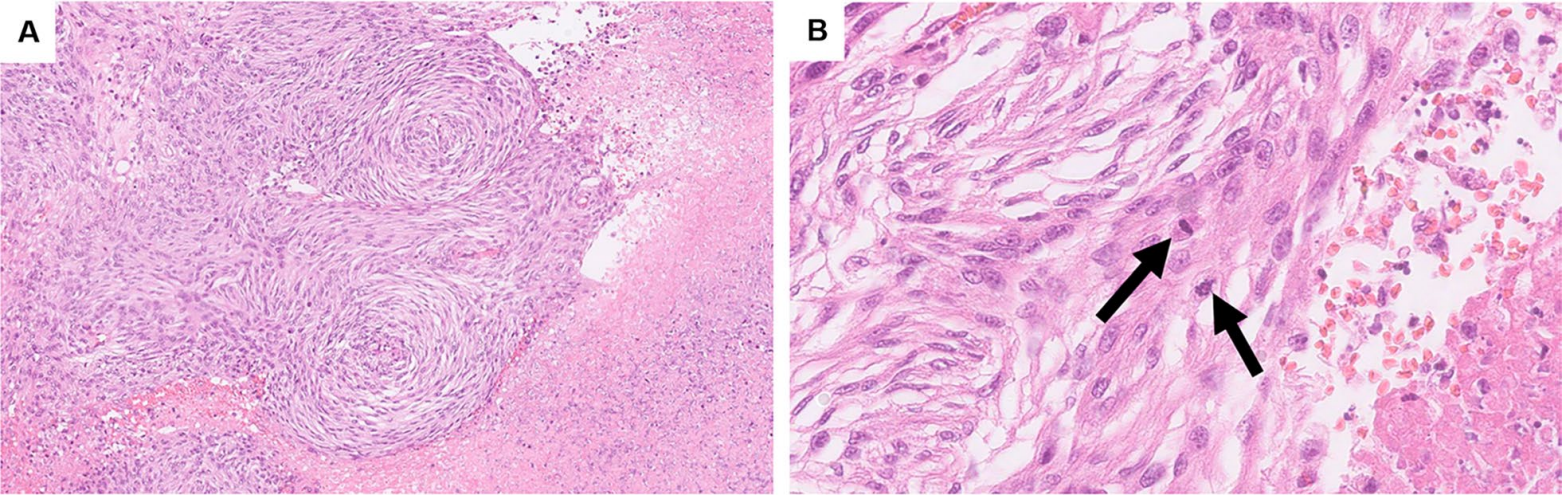
Histology of the resected specimen of malignant perineurioma. Spindle-shaped tumor cells proliferate in fascicular and whorl pattern with necrosis (**A** H&E, ×100). Tumor cells have uniform round-to-oval nuclei with indistinct nucleoli and mitotic figures (arrows) (**B** H&E, ×400)

**Fig. 4:**
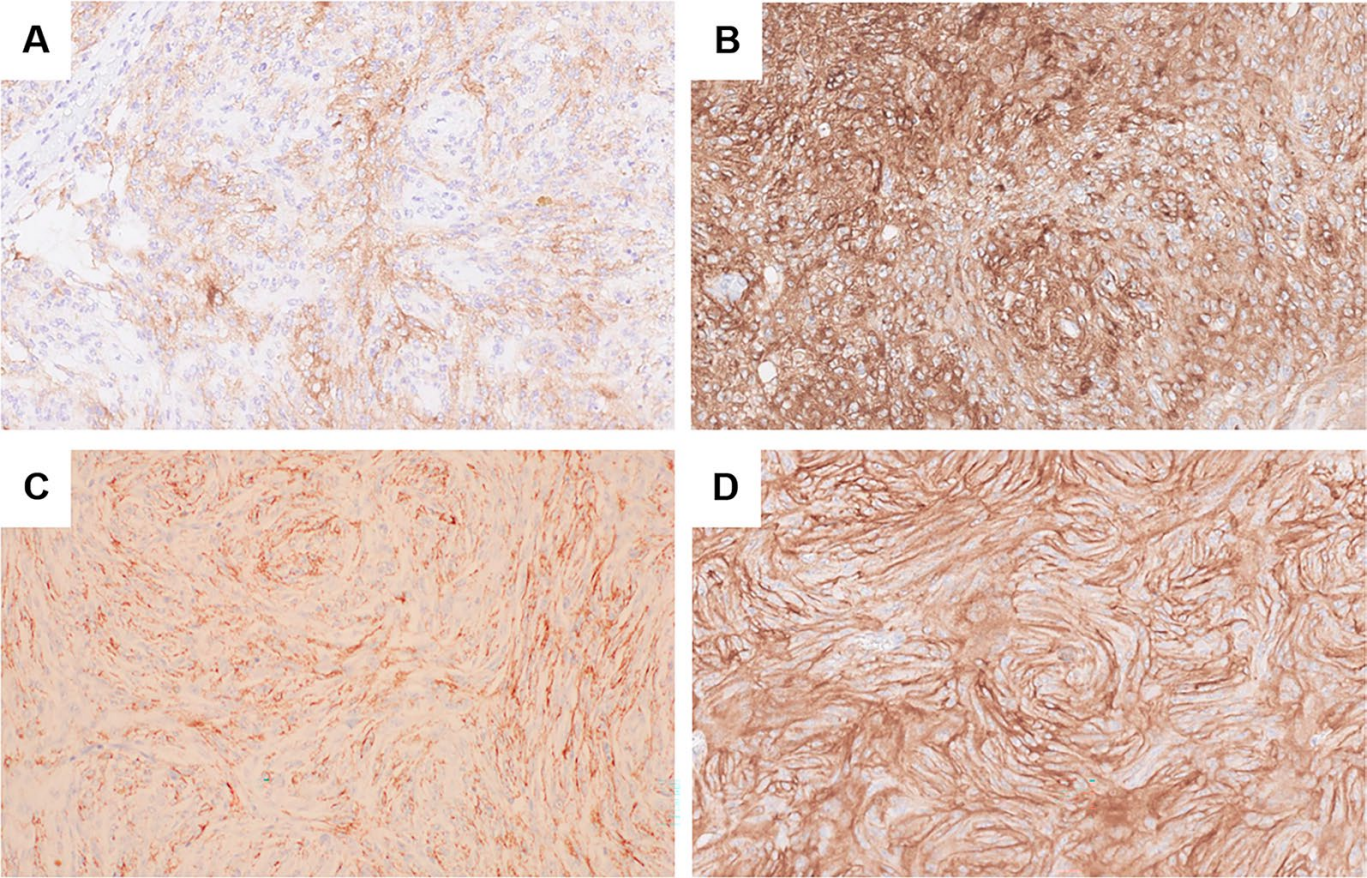
Immunohistochemical staining of the resected specimen at ×200 magnification. The tumor was positive for EMA (**A**), GLUT-1 (**B**), claudin-1 (**C**), and SSTR2A (**D**)

**Fig. 5:**
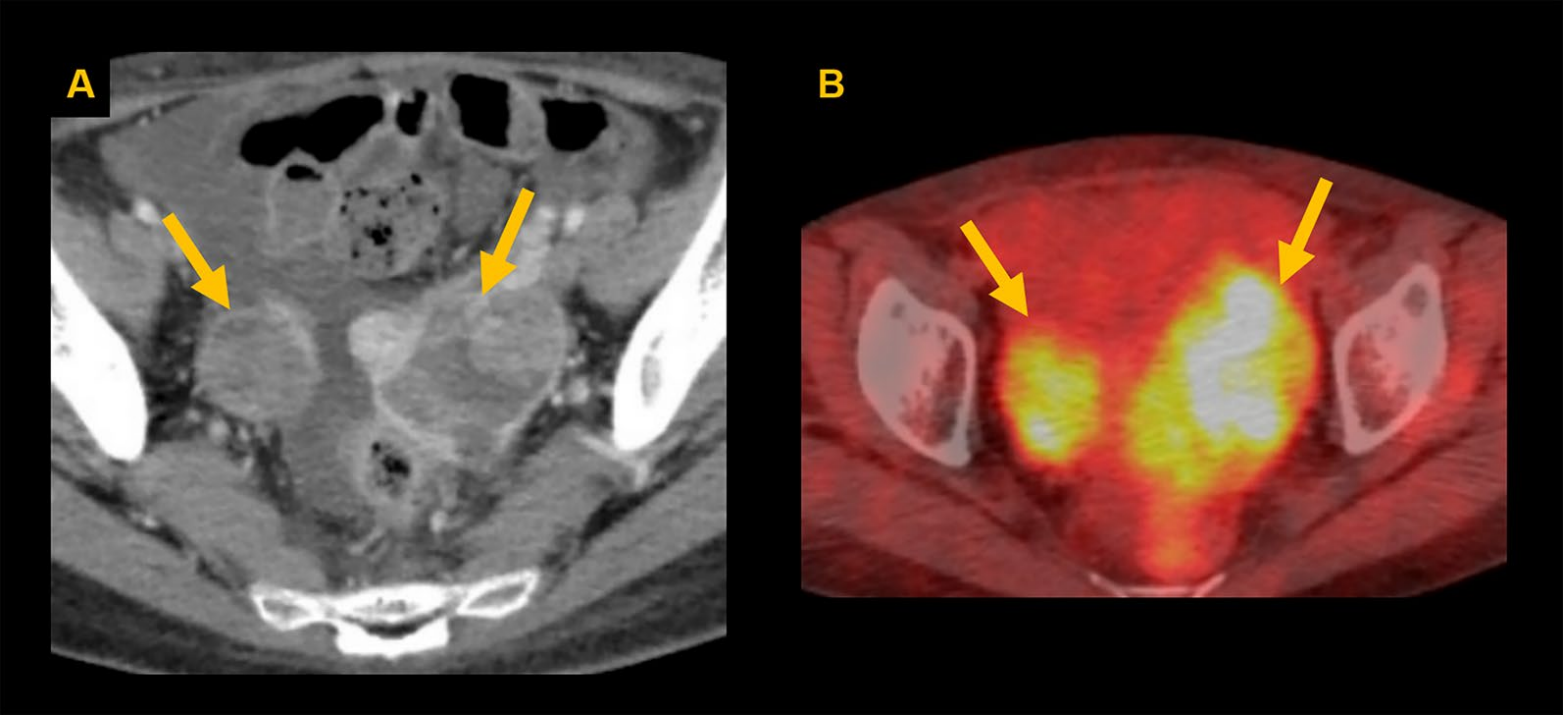
Early postoperative relapse. Contrast-enhanced CT images at 20 days postoperatively (**A**) shows bilateral pelvic masses (arrows) and FDG PET/CT images at 50 days postoperatively (**B**) confirmed their rapid growth with increased FDG uptake (arrows)

A 51-year-old female presented with abdominal distension and fatigue that had worsened in the past 1 year and was diagnosed with a retroperitoneal tumor. There was no history of tumors, and no family history suspected of hereditary tumors. On physical examination, a palpable mass was noted from the right subcostal area to the epigastric region. Blood tests revealed impaired renal function due to inferior vena cava (IVC) obstruction caused by the tumor. Tumor markers were negative. Contrast-enhanced computed tomography (CT) of the trunk showed a hemorrhagic retroperitoneal tumor measuring 22 × 9 × 15 cm, which had increased in size from 20 × 9 × 9 cm over the past 2 months, compressing IVC and portal vein to the right abdominal side (Fig. 1A). Enlarged para-aortic lymph nodes were also noted (Fig. 1B). Whole-body 18F-fluorodeoxyglucose positron emission tomography/CT (FDG PET/CT) showed increased FDG uptake with SUVmax 8.8 in the retroperitoneal tumor but no distant metastases. Endocrinological assessments were unremarkable, ruling out pheochromocytoma based on normal levels of catecholamines and metanephrines in both blood and 24-h urine. 123I-meta-iodobenzylguanidine (MIBG) scintigraphy revealed no abnormal accumulation, excluding paraganglioma or sympathetic nerve tumors. Considering the possibility of gastrointestinal stromal tumor (GIST) or other tumors responsive to drug treatment, a needle biopsy was performed preoperatively, revealing spindle-shaped sarcoma cells which raised dedifferentiated liposarcoma as a differential diagnosis and proceeded with resection of the retroperitoneal tumor. The tumor was extensively adherent to the left liver and IVC (Fig. 2A), and resected en bloc. Combined resection of the left liver and IVC was achieved without using extracorporeal circulation or IVC reconstruction, because the patient had double IVCs which prevented venous congestion of the kidneys and lower limbs. A small, disseminated tumor was also identified and retrieved. The blood loss was 17,490 mL. She developed a Grade B biliary leakage which required drainage and was discharged on the 35th day after surgery. The resected specimen was hemorrhaged and necrotic, with white lobulated nodules and edematous changes at the margins (Fig. 2B). There was no evidence of tumor invasion into liver and IVC. Microscopic examination revealed a spindle-shaped tumor cells proliferating in a fascicular and whorl pattern (Fig. 3A), and having round-to-oval nuclei with indistinct nucleoli (Fig. 3B). Necrosis (< 50%) and increased mitotic activity of 12 per 2mm^2^ suggested malignancy. Immunohistochemical staining demonstrated the tumor was positive for EMA (Fig. 4A), GLUT-1 (Fig. 4B), claudin-1 (Fig. 4C), and SSTR2A (Fig. 4D). The positivity for EMA, GLUT-1, and claudin-1 suggested differentiation toward perineurium [1], and malignant perineurioma was diagnosed. Of note, the tumor had no connection to the spinal leptomeninges. Negative staining for MDM2 and CDK4, CD34 and STAT6, and c-kit and DOG1 led to the exclusion of dedifferentiated liposarcoma, solitary fibrous tumor (SFT), and GIST, respectively [1, 8]. S100 protein was negative and H3K27e3 was retained. Cancer gene panel testing of the retroperitoneal tumor sample showed a base substitution in *ARID1A* (splice site 2251+2T>G), duplication of exons 5–31 in *NOTCH2*, frameshift mutation in *MSH6* (D387fs*4), and deletion of exons 5–9 in *NF2*, none of which were treatable target in the present case. Twenty days postoperatively, a contrast-enhanced CT of the trunk revealed masses in the bilateral pelvic walls (Fig. 5A), which were diagnosed to be peritoneal dissemination. Whole-body FDG PET/CT at 50 days postoperatively showed a short-term enlargement and increased uptake of the pelvic masses (Fig. 5B). Due to the rapid enlargement of tumors in both pelvic walls, accompanied by increased abdominal distension and worsening bilateral lower leg edema, palliative radiotherapy was performed from 70 days postoperatively. However, the disease progression could not be controlled, and the patient died 6 months postoperatively.

## Discussion

Soft tissue sarcomas of the retroperitoneum are predominantly liposarcoma and leiomyosarcoma, with SFT and MPNST being less common, but malignant perineurioma is extremely rare and its frequency is uncertain [9]. Malignant perineurioma occasionally has very aggressive characteristic with poor survival outcomes with 5-year overall survival of 67% and 5-year recurrence-free survival of 33% [2–6]; however, this tumor has not been fully studied and effective treatment has not been well established. We report a second case of malignant perineurioma derived from the retroperitoneum which progressed rapidly. The cancer gene panel testing identified several genetic alterations, including a *NF2* mutation, but no molecular target drug was found, and the patient died 6 months after surgery.

Perineuriomas and ectopic meningiomas are embryologically, histologically, and immunohistologically similar and are indistinguishable in a non-hereditary soft tissue origin case [10], and thus, such a distinction can be considered semantic. In our case, the immunohistochemical staining demonstrated the tumor was positive for SSTR2A in addition to EMA, which could be viewed as a sign favoring differentiation toward meninges [1], although the patient had no lesion in contact with meninges in the head, neck, or spine. Tumors that are histopathologically considered to be of meningeal origin but arise in tissues where meninges are not normally present are called as ectopic meningiomas [1, 11]. The frequency of ectopic meningiomas is estimated to be less than 1% of all meningiomas [12]. To the best of our knowledge, only 16 cases of perineuriomas or ectopic meningiomas arising in the retroperitoneum, including one case of malignant perineurioma, have been reported so far [7, 13–24], as summarized in Table 1. Resection was performed in all patients. Tumor recurrence was observed in three of 16 cases and treated with chemotherapy (details unknown) in one and re-resection and irradiation in one.

**Table 1:** Reported cases of ectopic meningioma or perineurioma arising from the retroperitoneum

Report	Diagnosis	Age (yr)/sex	Size (cm)	Treatment	Follow-up (mo)
Huszar*_*1996	Ectopic meningioma	25/F	22 × 13 × 16	Primary: resection	Rec, 6
				Relapse: chemotherapy	DRD, 18
Mao*_*2014	Ectopic meningioma	53/M	11 × 9 × 18	Primary: resection	Rec, 30
				Relapse: resection + radiation therapy	ANED, 6
Ramlagun_2018	Ectopic meningioma	30/M	1.1	Resection (left adrenal)	ANED, 12
García*_*1998	Perineurioma	7/F	3	Resection (right kidney)	NA
Hirose_1998	Malignant perineurioma	83/M	30 × 21 × 15	Resection (retroperitoneum)	Rec
					DUD, 28
Balarezo_2003	Perineurioma	14/F	14.8 × 8.8 × 5.6	Resection (retroperitoneum)	ANED, 48
Hornick_2005	Perineurioma	59/F	14	Resection (retroperitoneum)	NA
	Perineurioma	57/M	2.7	Resection (retroperitoneum)	ANED, 36
	Perineurioma	68/F	12	Resection (presacral)	ANED, 24
Rampisela_2009	Perineurioma	28/F	3	Resection (right adrenal)	ANED, 18
Yasumoto_2010	Perineurioma	63/M	4	Resection (retroperitoneum)	ANED, 16
Huang*_*2012	Perineurioma	25/M	14 (left), 10 (left), 6 (right)	Resection (bilateral kidney)	ANED, 24
Saha*_*2012	Perineurioma	35/F	15 × 15	Resection (pancreas)	NA
Gan*_*2014	Perineurioma	40/M	12	Resection (right transplanted kidney)	NA
Ma_2021	Perineurioma	49/F	6.5	Resection (left kidney)	ANED, 66
	Perineurioma	42/M	12	Resection (left kidney)	ANED, 24
Our case	Malignant perineurioma	51/F	22 × 18 × 18	Primary: resection	Rec, < 1
				Relapse: radiation therapy	DRD, 6

*NA* not available, *ANED* alive with no evidence of disease, *DUD* died of unrelated disease, *Rec* recurrence, *DRD* disease-related death

Treatments for malignant perineuriomas have been developed in conjunction with those for MPNST [9], as both malignant perineurioma and MPNST have origins anatomically very close within the peripheral nerve. Resection is the primary choice of treatment for malignant perineuriomas [18]. Some studies advocated the use of preoperative chemotherapy for malignant perineurioma, for which anthracycline regimens such as epirubicin–ifosfamide chemotherapy are more effective than etoposide–ifosfamide regimen in terms of significantly better disease-free survival (hazard ratio 2.38) [25]. For the treatment of recurrent or metastatic perineurioma, doxorubicin–ifosfamide chemotherapy may be recommended, providing better median progression-free survival (PFS) of 26.9 weeks compared with anthracyclines alone, CYVADIC therapy with cyclophosphamide, vincristine, adriamycin, and dacarbazine and ifosfamide alone (median PFS of 17, 10.4, and 9.4 weeks, respectively) [26]. Postoperative irradiation is sometimes used for malignant perineuriomas to decrease the chance of recurrence [27].

Cancer gene panel testing of the present retroperitoneal tumor revealed a deletion of exons 5–9 in *NF2*, in accordance with previous reports revealing *NF2* mutations in both meningiomas and perineuriomas [28, 29]. *NF2* encodes a tumor suppressor, moesin–ezrin–radixin-like protein (Merlin), which regulates cell survival and proliferation and is also involved in intercellular adhesion [30]. Merlin-deficient malignant mesothelioma showed, in a preclinical study, a strong synthetic lethal relationship between Merlin and FAK, which also regulating cell survival, proliferation, invasion, and cancer stem cell regeneration [31, 32]. Since intercellular adhesion is reduced in Merlin-deficient cells, their survival and proliferation are highly dependent on integrin/FAK signaling through cell–extracellular matrix contact [31]. Based on promising results of a phase II trial evaluating FAK inhibitors for recurrent or advanced *NF2* mutation-positive intracranial meningiomas, which demonstrated PFS of 83% for WHO grade 1 and 33% for grade 2/3 patients at 6 months [33], further evaluation of FAK inhibitor for malignant perineurioma and ectopic meningioma is desired.

## Conclusions

We present a rare case of malignant perineurioma derived from the retroperitoneum with very aggressive characteristics. The identification of an *NF2* mutation through cancer gene panel testing provides valuable insights into potential therapeutic strategy.

## Data Availability

Not applicable.

## Abbreviations

IVC: Inferior vena cava
CT: Computed tomography
MRI: Magnetic resonance imaging
FDG PET: 18F-fluorodeoxyglucose positron emission tomography
MIBG: 123I-meta-iodobenzylguanidine
GIST: Gastrointestinal stromal tumor
SFT: Solitary fibrous tumor
MPNST: Malignant peripheral nerve sheath tumor
PFS: Progression-free survival
Merlin: Moesin–ezrin–radixin-like protein
